# A useful method of identifying of miRNAs which can down-regulate Zeb-2

**DOI:** 10.1186/1756-0500-6-470

**Published:** 2013-11-18

**Authors:** Shigeyoshi Oba, Takayuki Mizutani, Etsu Suzuki, Hiroaki Nishimatsu, Masao Takahashi, Yousuke Ogawa, Kenjiro Kimura, Yasunobu Hirata, Toshiro Fujita

**Affiliations:** 1 Department of Nephrology and endocrinology, University of Tokyo School of Medicine, 113-8655, 7-3-1, Hongo, Bunkyo-ku, Tokyo, Japan; 2 Department of Molecular Pathology, Tokyo Medical University, Tokyo, Japan; 3 Institute of Medical Science, St. Marianna University School of Medicine, Kawasaki, Japan; 4 Department of Urology, University of Tokyo School of Medicine, Tokyo, Japan; 5 Department of Cardiovascular disease, University of Tokyo School of Medicine, Tokyo, Japan; 6 Division of Nephrology and Hypertension, St. Marianna University School of Medicine, Kawasaki, Japan; 7 Division of Clinical Epigenetics, Research Center for Advanced Science and Technology, The University of Tokyo, Tokyo, Japan

## Abstract

**Background:**

Although identification of the target mRNAs of micro RNAs (miRNAs) is essential to understanding their function, the low complementarity between miRNAs and their target mRNAs has complicated this process. In this study, we sought to identify miRNAs which reduce the expression of the transcription factor Zeb-2, a transcriptional repressor of E-cadherin which is known to be down regulated by members of the miR-200 family (miR-200a,b,c miR-429, and miR-141).

**Findings:**

We first used a computational target predicting system to identify 82 candidate miRNAs which bound the 3′UTR region of the *Zeb-2* mRNA. Of these 82 miRNAs, precursors for 51 were available in our miRNA precursor library. Pre-miR™ Precursor Molecules for these 51 miRNAs were co-transfected into NIH3T3 cells with a luciferase reporter vector containing the 3′UTR region of the *Zeb-2* mRNA. Seven miRNAs (miR-141, mi-183, miR-200a, miR-200b, miR-200c, miR-429 and miR-666-5p) were shown to down-regulate luciferase activity and Western blotting analysis confirmed that Pre-miR™ Precursor Molecules for these seven miRNAs induced expression of E-cadherin and miScript target protector against miR-183 and miR-666-5p abrogated this effect. Moreover, an Anti-miR™ miRNA Inhibitor targeting miR-183 and miR-666-5p repressed expression of E-cadherin.

**Conclusions:**

We have established a method to identify miRNAs that bind the 3′UTR region of the Zeb-2 mRNA and that induce expression of E-cadherin, possibly by down-regulating the expression of Zeb-2. Our method may be more widely applicable for identifying miRNAs that bind target mRNA 3′UTR regions and down-regulate the expression of proteins encoded by these mRNAs.

## Background

MicroRNAs (miRNAs) are 22-nucleotide (nt) endogenous non-coding RNAs that exhibit a high degree of structural and functional conservation throughout different species. miRNAs are initially synthesized as long primary transcripts that are subsequently processed into -70-nt stem-loop pre-microRNAs by Drosha endonucleasae [1] and transported out of the nucleus by exportin 5 [2]. Dicer then processes these pre-microRNA in the cytoplasm to yield the -22-nt mature miRNAs [3]. Binding of miRNA to target mRNAs with perfect or near perfect complementarity has been shown to induce mRNA degradation, whereas imperfect complementarity reportedly induces translational regression. In this context, 7–8 nt sequence at the 5′ end of the miRNA sequence, known as the seed sequence, is known to be critical for efficient targeting.

miRNAs have been implicated in regulating complex physiological processes such as embryogenesis [4], organ development [5], and oncogenesis [6,7], and we have recently demonstrated their role in the pathology of kidney diseases [8]. To date over 2000 miRNAs have been identified in human, although the functional roles of the vast majority remain unknown.

Whereas identifying the target mRNAs of miRNAs is essential to understanding their biological roles, this has proven difficult due to the imperfect complementarily between miRNAs and their target mRNAs. cA recently described method for identifying miRNA target mRNAs using exogenously tagged Ago2 [9] has proven effective, but has a complicated protocol and carries a high rate of identification of artifactual mRNAs such as mitochondrial mRNAs. Other approaches have used microarray analysis to identify miRNAs targeting disease-related proteins. Global expression profiling of cancer cell lines overexpressing miR-16, for example, identified 27 candidate targets of this miRNA, of which three (MAP7, PRDM44 and CDS2) were experimentally validated [10]. In general however, such methods have proven unreliable for detection of disease-related miRNA target genes. Here, we set out to establish a reliable method for identifying miRNAs which down-regulate the expression of a specific target protein.

Many miRNAs have been shown to bind to the 3′UTR region of their target mRNA. Previous studies have demonstrated miRNA-mediated repression of the translation of a luciferase reporter gene to which the 3′UTR coding region of the target mRNA has been spliced [11]. In this proof-of-principle study, we used a similar method to identify miRNAs which down-regulated expression of the E-box-binding zinc-finger transcription factor Zeb-2, which has previously been shown to be repressed by members of the miR-200 family (miR-200a,b,c miR-429, and miR-141) [11-13].

We first established dual luciferase reporter vector containing the 3′UTR coding region of *Zeb-2* mRNA spliced to the 3′ end of the luciferase coding region. We then transfected cultured cells with this reporter vector in 96-well plates and 48 hours later transfected cells with a series of miRNA precursors. Luciferase activity was assayed after 72 hours later to identify miRNAs which bound the 3′UTR of Zeb-2 and subsequently reduced luciferase expression. This method proved cumbersome however, prompting us to use a computational target predicting system to narrow down the number of screened miRNAs to a figure (<100 miRNAs) that was manageable, which a previous study had shown was likely to yield true functional miRNAs (data not shown). We anticipate that our new method will be valuable for future characterization of miRNA function.

## Materials and methods

### Prediction of miRNAs by computational target predicting system

To detect candidate miRNAs targeting Zeb2, we first evaluated a series of miRNA precursors. To narrow the screened miRNAs to below 100 miRNAs, we used a computational target predicting system (miRanda) containing updated sequences for all known miRNAs [14-16]. Cutoff scores for selection of candidate miRNAs were < -20.0 for energy and >120 for binding.

### ZEB2 3′-UTR luciferase reporter vector

We designed a dual luciferase reporter vector containing the 3′UTR coding region of Zeb-2 mRNA spliced to the 3′end of the luciferase coding region. Firstly, the 3′-UTR for *ZEB2* was PCR-amplified from mouse genomic DNA. The PCR primers used to amplify the Zeb-2 3′-UTR were 5′-CAGTTCAGCCAAGACAGAGT-3′ (forward) and 5′-TTCGAGCATGGTCATTTTC-3′UTR (reverse). The amplified 3′-UTRs was cloned downstream of the firefly luciferase coding region in the pGL3-promoter luciferase reporter vector (Clontech). Sequence analysis confirmed the accuracy of the PCR procedure.

### miRNA precursor library

In this study we used the Pre-miR™ miRNA Precursor Library - Mouse V3 (Ambion co.).

Pre-miR™ miRNA Precursor Molecules are small, chemically modified double-stranded RNA molecules designed to mimic endogenous mature miRNAs. The Pre-miR™ miRNA Precursor Library–Mouse V3 consists of 379 miRNA mimics corresponding to 379 mouse mature miRNAs cataloged in version 9.2 of the miRBase Sequence Database.

### Cell culture

NIH3T3 and HK-2 cell lines were provided by the RIKEN BRC through the National Bio-Resource Project of MEXT, Japan. NIH3T3 cells were cultured in Dulbecco’s Modified Eagle Medium (DMEM) containing 10% bovine serum. HK-2 cells were grown in DMEM supplemented with 5% fetal bovine serum. Cells were routinely cultured at 37°C in a humidified atmosphere of 95% air-5% CO_2_.

### Transfection and luciferase assays

NIH3T3 cells were seeded in 96 well plate (Sterilin UK). 24 hr later, 0.1 μg of reporter plasmid was transfected using the Lipofectamine LTX system (Invitrogen), according to the manufacturer’s protocols. To assess the effect of miRNAs on reporter activity, 2.5 pmol of synthetic Pre-miR™ miRNA Precursor Molecules and Pre-miR™ miRNA Precursor Molecules-Negative Control (Ambion co.) were transfected 48 hours later using the Lipofectamine RNAiMax system (Invitrogen), according to the manufacturer’s protocols. Cells were lysed after 24 hours in 40 μl passive lysis buffer (Promega). Measurements were performed using the Promega luciferase assay system and the GloMax 96 Microplate Luminometer (Promega). Each sample was measured in four replicates. The luciferase activity of each lysate was normalized to the luciferase activity of the Pre-miR™ Precursor Molecules-Negative control.

### Western blotting of E-cadherin

Western blotting analysis of E-cadherin was performed in NIH3T3 cells. To investigate the effect of miRNAs on Zeb-2 levels, NIH3T3 cells were transfected with 20 pmol/ml Pre-miR™ Precursor Molecules and Anti-miR™ miRNA Inhibitor (Ambion) using the Lipofectamine RNAiMax system (Invitrogen). To show a direct miRNA-mRNA interaction at the predicted target sites, NIH3T3 cells were transfected with 20 pmol/ml miRNA precursors and 300 pmol miScript target protector (Qiagen) against miR-183 or miR-666-5p simultaneously. After 24 hours the cell lysate was extracted and analysed by Western blotting using a mouse monoclonal anti-E-cadherin antibody (BD Bioscience). as previously described [8]. To confirm that the same amount of protein was investigated, the expression of beta-actin was also investigated.

### Effect of miRNAs on epithelial and mesenchymal transition

To examine the effect of microRNAs to epithelial mesenchymal transition, we performed morphological observation and fluorescent staining of filamentous actin (F-actin) in transfected cells. Normal human kidney HK-2 cells (purchased from RIKEN) were transfected with Pre-miR™ miRNA Precursor Molecules for miR-183or miR-666-5p. 24 hours later 4 ng/ml TGF-beta was added and another 24 hours later, cells were observed under phase contrast microscope. Cells were then fixed with 4% paraformaldehyde and incubated with Rhodamine-conjugated Phalloidin (CHEMICON) for 30 min, before observation under a laser microscope.

### Statistical analysis

All data are reported as mean ± SD. When comparisons were made between two different groups, statistical significance was determined using the Student’s *t*-test.

## Findings

## Results

### Prediction of Zeb-2 mRNA 3’UTR-binding miRNAs

We identified in silico 82 candidate miRNAs (Table 1) which were predicted to directly bind the 3′UTR of the Zeb-2 mRNA and regulate expression of Zeb-2. These candidates included all miRNAs, namely miR-141, miR-200a,b,c and miR-429, previously shown to directly regulate Zeb-2.

**Table 1:** **Candidate miRNAs which can bind 3′UTR of *Zeb-2 *** **mRNA**

mmu-miR-1187	mmu-miR-2142	mmu-miR-467d
mmu-miR-1188	mmu-miR-22	mmu-miR-469
mmu-miR-124	mmu-miR-23a	mmu-miR-470
mmu-miR-125a-3p	mmu-miR-23b	mmu-miR-471
mmu-miR-136	mmu-miR-27a	mmu-miR-540-5p
mmu-miR-138	mmu-miR-28	mmu-miR-541
mmu-miR-141	mmu-miR-298	mmu-miR-547
mmu-miR-152	mmu-miR-301b	mmu-miR-574-5p
mmu-miR-155	mmu-miR-320	mmu-miR-666-5p
mmu-miR-183	mmu-miR-324-3p	mmu-miR-669c
mmu-miR-1896	mmu-miR-328	mmu-miR-669 g
mmu-miR-1906	mmu-miR-33	mmu-miR-674
mmu-miR-1927	mmu-miR-342-3p	mmu-miR-680
mmu-miR-1931	mmu-miR-34a	mmu-miR-686
mmu-miR-1933-5p	mmu-miR-379	mmu-miR-691
mmu-miR-1935	mmu-miR-380-3p	mmu-miR-693-3p
mmu-miR-1941-5p	mmu-miR-409-5p	mmu-miR-697
mmu-miR-1948	mmu-miR-429	mmu-miR-698
mmu-miR-1958	mmu-miR-448	mmu-miR-706
mmu-miR-1960	mmu-miR-449a	mmu-miR-708
mmu-miR-1962	mmu-miR-449b	mmu-miR-712
mmu-miR-1966	mmu-miR-449c	mmu-miR-742
mmu-miR-200a	mmu-miR-452	mmu-miR-872
mmu-miR-200b	mmu-miR-466b-5p	mmu-miR-873
mmu-miR-200c	mmu-miR-466c-5p	mmu-miR-880
mmu-miR-205	mmu-miR-466e-5p	mmu-miR-881
mmu-miR-20a	mmu-miR-467c	mmu-miR-96
mmu-miR-2138		

### Screening of miRNAs which can down-regulate the expression of Zeb-2

Of these 82 miRNAs, Pre-miR™ miRNA Precursor Molecules for 51which were available in our Pre-miR™ miRNA Precursor Library, were co-transfected into NIH3T3 cells along with a luciferase reporter vector containing the 3′UTR coding region of Zeb-2. Pre-miR™ miRNA Precursor Molecules for miR-141, miR-183, miR-200a, miR-200b, miR-200c, miR-429 and miR-666-5p down-regulated luciferase activity below 80% compared to control, (miR-141: 0.6 ± 0.106, miR183: 0.6 ± 0.106, miR-200a: 0.645 ± 0.031, miR-200b: 0.752 ± 0.163, miR-200c: 0.603 ± 0.03, miR-429: 0.633 ± 0.076) (Table 2).

**Table 2:** Relative luciferase activity of candidate miRNA

**Control**	**1 ± 0.06**
miR-124	1.15 ± 0.025
miR-136	1.17 ± 0.018
miR-138	0.952 ± 0.16
miR-141	0.6 ± 0.106
miR-152	0.919 ± 0.044
miR-155	0.934 ± 0.183
miR-183	0.769 ± 0.022
miR-200a	0.645 ± 0.031
miR-200b	0.752 ± 0.163
miR-200c	0.603 ± 0.03
miR-205	0.927 ± 0.017
miR-20a	0.963 ± 0.017
miR-22	0.954 ± 0.157
miR-23a	0.896 ± 0.038
miR-23b	0.904 ± 0.051
miR-27a	0.984 ± 0.156
miR-28	1.071 ± 0.038
miR-298	0.874 ± 0.08
miR-301b	1.032 ± 0.119
miR-320	0.964 ± 0.007
miR-324-3p	1.194 ± 0.031
miR-328	1.202 ± 0.016
miR-33	1.051 ± 0.125
miR-342-3p	0.854 ± 0.086
miR-34a	0.917 ± 0.043
miR-379	1.062 ± 0.140
miR-380-3p	0.958 ± 0.016
miR-429	0.633 ± 0.076
miR-448	0.931 ± 0.133
miR-449a	1.027 ± 0.023
miR-449b	1.022 ± 0.020
miR-452	0.826 ± 0.053
miR-469	1.096 ± 0.112
miR-470	0.948 ± 0.062
miR-471	0.814 ± 0.059
miR-541	0.985 ± 0.124
miR-547	0.934 ± 0.04
miR-666-5p	0.762 ± 0.022
miR-669c	0.965 ± 0.117
miR-674	0.993 ± 0.015
miR-680	1.148 ± 0.025
miR-686	1.176 ± 0.009
miR-691	1.018 ± 0.202
miR-693-3p	0.889 ± 0.005
miR-697	0.979 ± 0.055
miR-698	1.002 ± 0.213
miR-706	1.037 ± 0.037
miR-708	0.983 ± 0.042
miR-712	0.821 ± 0.216
miR-742	0.976 ± 0.047
miR-96	0.927 ± 0.033

### Prediction of target sites of the identified miRNAs in the Zeb-2 3′UTR

**Figure 1:**
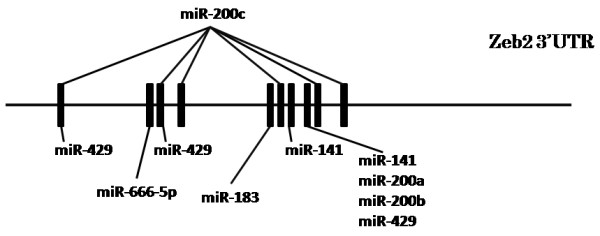
**Schematic of putative detected miRNAs target sites for Zeb-2 3′UTR.** Black boxes represent target sites for each miRNAs.

The miRanda computational target predicting system identified potential target sites for the candidate miRNAs in the Zeb-2 3′UTR (Figure 1). miR-183, miR-200a, miR-200b and miR-666-5p have at least one potential target site, while miR-141 has two, miR-429 has three and miR-200c has six.

### Western blotting of E-cadherin

**Figure 2:**
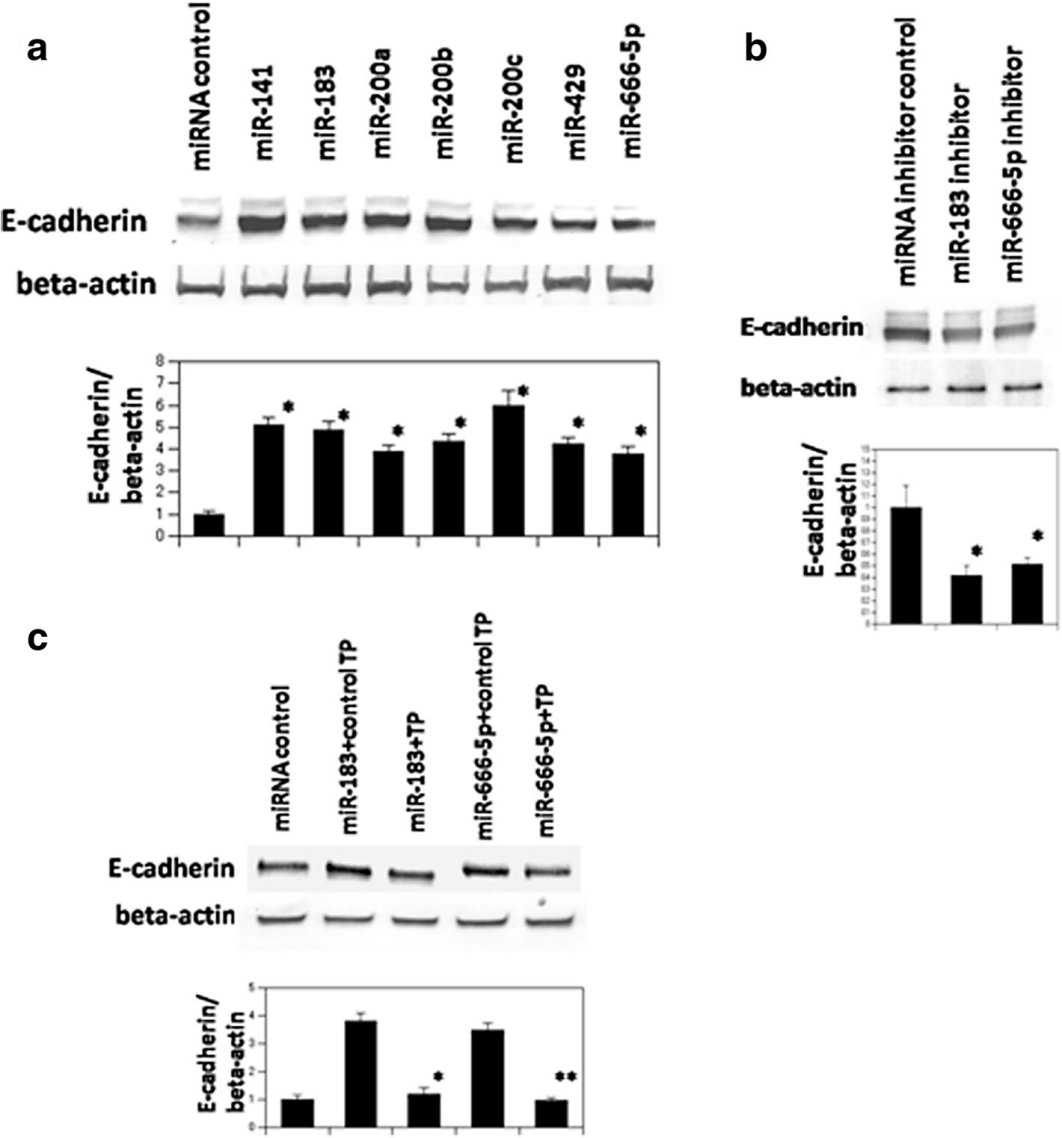
**Western blotting of E-cadherin. (a)** Western blotting of E-cadherin in NIH3T3 cells transfected with control miR or Pre-miR™. Precursor Molecules individually. *p < 0.05, compared to cells transfected with Pre-miR™ miRNA Precursor Molecules-Negative Control. **(b)** Western blotting of E-cadherin in NIH3T3 cells transfected with Anti-miR™ miRNA Inhibitor against miR-183 and miR-666-5p. *p < 0.05, compared to cells transfected with Anti-miR™ miRNA Inhibitor-negative control. **(c)** Western blotting of E-cadherin in NIH3T3 cells transfected with Pre-miR™ Precursor Molecules for miR-183 or miR-666-5p precursors and each Script Target Protector. *p < 0.05, compared to cells transfected with Pre-miR™ Precursor Molecules for miR-183 and miScript target protector-negative control, **p < 0.05, compared to cells transfected with re-miR™ Precursor Molecules for miR-666-5p and miScript target protector-negative control. The data are mean ± S.D. of triplicates and expression levels of E-cadherin were normalized to beta-actin with further normalization to the negative control.

Zeb-2 has been shown to be a transcriptional repressor of E-cadherin. Since we were unable to obtain an appropriate antibody for Zeb-2, we performed western blotting of E-cadherin as a surrogate marker for Zeb-2 expression. Western blotting analysis showed that Pre-miR™ Precursor Molecules for the seven miRNAs referred to above up-regulated expression of E-cadherin (Figure 2a), most likely due to their down-regulation of the expression of Zeb-2. Focusing on miR-183 and miR-666-5p, we next examined whether Anti-miR™ miRNA Inhibitor against miR-183 and miR-666-5p could down-regulate the expression of E-cadherin. Western blotting analysis confirmed that this was the case (Figure 2b). miScript target protectors are modified RNA oligonucleotides complementary to specific target sites and which do not bind other sequences [17]. Transfection of custum-desigend miScript target protectors against the predicted miR-183 and miR-666-5p target sites in the Zeb-2 3′UTR abrogated the effect of the Pre-miR™ Precursor Molecules for miR-183 and miR-666-5p (Figure 2c).

### Cluster analysis of seven miRNAs

**Figure 3:**
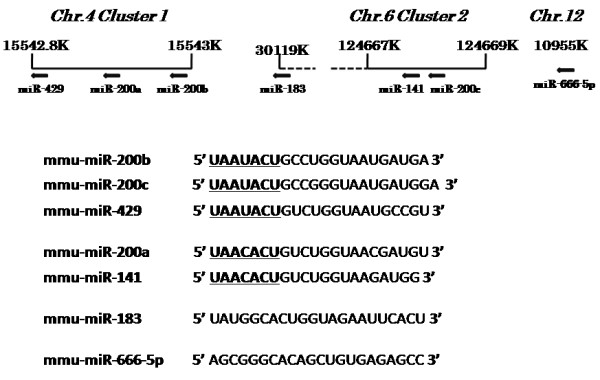
**Upper panel, schematic of chromosomal location of the detected miRNAs in the mouse genome.** Lower panel, sequence alignment of the detected miRNAs. Nucleotides 2–7, representing their seed sequences, are underlined.

The miR-200 family of miRNAs has been mapped to two separate clusters of less than 2000 bp each in the mouse genome (Figure 3 upper panel) [3]. The first cluster (Cluster 1) contains miR-200a, miR-200b and miR-429 and is located in chromosome 4. The second cluster (Cluster 2), containing miR-200c and miR-141, is located in a 500-bp region of chromosome 6. The two remaining miRNAs we identified, miR-183 and miR-666-5p, are located on chromosome 6 (30119 K) and chromosome 12 (110955 K) respectively. The five miR-200 family miRNAs located in the chromosome 4 and 6 clusters contain very similar seed sequences (Figure 3 lower panel) which are not shared with miR-183 and miR-666-5p.

### The effect to epithelial and mesenchymal transition

**Figure 4:**
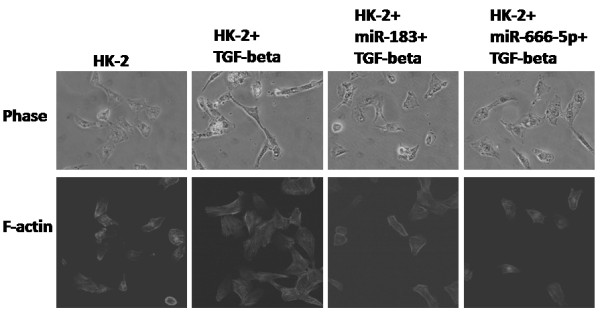
**TGF-beta induced epithelial mesenchymal transition to HK-2 cells which was inhibited by Pre-miR™ Precursor Molecules for miR-183 and miR-666-5p.** Phase contrast and Immunofluorescence staining of F-actin of HK-2 cells which was stimulated with 4 ng/ml TGF-beta and, Pre-miR™ Precursor Molecules for miR-183 and miR-666-5p.

Observation under phase contrast microscope revealed that the addition of Transforming growth factor (TGF)-β resulted in the appearance of the hallmarks of epithelial to mesenchymal transition, including a change in morphology from round compact shape to spindle shaped. This was accompanied by a rearrangement of actin filaments from a cortical to a stress-fiber pattern (Figure 3) [10]. Transfection of Pre-miR™ miRNA Precursor Molecules for miR-183 and miR-666-5p inhibited the appearance of these changes (Figure 4).

## Discussion

We report here a novel method of identifying miRNAs which bind to the 3′UTR region of the Zeb 2 mRNA and that up-regulate expression of E-cadherin. Several reports have shown that members of the miR-200 family (miR-200a,b,c, miR-141 and miR-429) inhibit epithelial mesenchymal transition (EMT) through direct targeting of *ZEB1* and *ZEB2*, which encode transcriptional repressors of E-cadherin in kidney tubular cells [4], breast cancer cells [5], and mammary epithelial cells [3]. Recent reports have indicated that a double-negative feedback loop between *ZEB1*, *ZEB2* and miRNA-200 family members regulates EMT in kidney tubular cells [18].

In this report, besides confirming down-regulation of Zeb-2 by member of the miR-200 family, we have shown that miR-183 and miR-666-5p can also down-regulate Zeb-2. Using a novel screening method, we demonstrated that while miR-23a, 298, 342-3p,452, 471, 693-3p, 712 down-regulated luciferase activity (above 80% compared to control), they had no effect on E-cadherin levels, and excluded them from further analysis.

While no target of miR-666-5p has been previously reported, several studies have shown that miR-183 is up-regulated in prostate carcinoma and breast cancer [19,20]. Given that EMT has been known to be associated with tumorigenesis, it may be that up-regulation of miR-183 inhibits EMT in tumor tissue.

Our study contains another important new finding. Many computational target prediction systems generate large numbers of candidate miRNAs that potentially down-regulate the expression of the proteins encoded by these mRNAs. While we can not know in fact how many miRNAs down-regulate the expression of a given protein, based on our results, only about seven miRNAs can up-regulate expression of E-cadherin, most likely due to their down-regulation of the expression of Zeb-2. We have not investigated all currently known miRNAs, however we can infer that the number of miRNAs that can down-regulate a single protein is limited.

Identifying the target mRNAs of miRNAs is essential to understanding the cellular regulatory networks in which miRNAs are involved, but due to the low complementarity between miRNAs and their target mRNAs, only a few mammalian target mRNAs have been identified. To ameliorate this situation, a variety of prediction algorithms, such as miRanda [15,21], TargetScan [14,22,23], Pic Tar [24], RNA22 [25], RNA hybrid [26], PITA [27], EiM Mo [28] and DIANA [29], have been developed. These algorithms use a spectrum of parameters, including binding energy of the duplex structure, evolutionary conservation of the target site and secondary structure of 3′UTR. Despite this, they generate multiple false positive candidates and, accordingly, experimental verification of predicted miRNA-mRNA interactions must be performed. High-throughput methods successfully used in validation of miRNA target sites, including microarray and proteomic analysis, are based on measuring changes in the mRNA and protein level in response to miRNA introduction [10]. However, they cannot identify microRNAs which down-regulate a specific target protein and reporter assay such as that used in this study are required to validate candidate miRNAs routinely and with high specificity.

**Figure 5:**
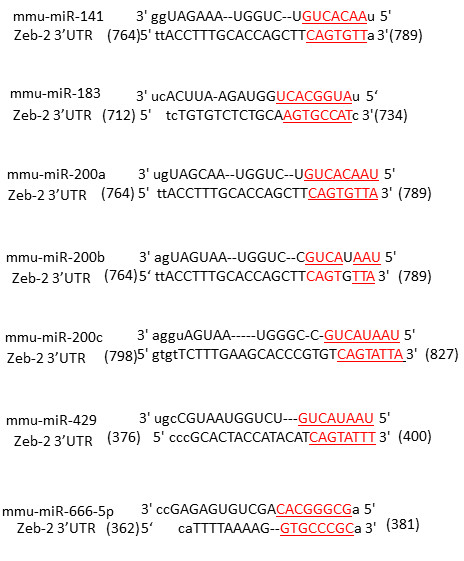
**Sequence alignment of microRNAs.** Their seed sequences are underlined.

Our study contains one more important new finding. Until now, we have been unable to determine the number of seed sequence matches needed for the binding of miRNAs to target mRNA 3′UTR. In this reports, we identified seven miRNAs which can directly regulate the expression of Zeb-2. The number of seed sequence matches for these miRNAs were eight (miR-200a, miR-200c, miR-183, miR-429 and miR-666-5p) and seven (miR-141 and miR-200b) (Figure 5), indicating that at least seven seed sequence matches are desirable for a miRNA to bind to a target mRNA 3′UTR. This information is useful for the identification of miRNAs which can directly regulate disease related proteins.

Immunoprecipitation of RNA induced silencing complex (RISC)-associated mRNA requires a large amount of starting material. Using a modification of this protocol, Hayashida et al. constructed an efficient and convenient system for analyzing the mRNA content of RISC [2], and identified mRNA targets of individual miRNAs. The RISC complex however, contains multiple RNA species. For example, Hayashida et al. showed that while almost all of the cDNA recovered from RISC was miRNA (94%), the remainder contained rRNA and tRNA clones (3% and 2%, respectively) and cDNA clones (1%) with hits in genomic DNA sequences. These facts indicate the difficulty of identifying target mRNAs in the RISC complex.

We set out in this study to develop a new method for identifying target mRNAs for miRNAs. The originality of our method is that it is based upon a functional endpoint, namely down-regulation of a target protein. Using our method we will be in a position to identify miRNAs which can bind mRNA 3′UTR regions and down-regulate the expression of the encoded proteins. In future, we hope that the joint efforts of researchers all over the world will enable us to establish a database of miRNAs which can down-regulate the expression of specific protein targets.

## Competing interests

The authors declare that they have no competing interests.

## Authors’ contributions

SO: performed and analysed experiments, and wrote the manuscript. TM: performed prediction of miRNAs by computational target predicting system. YO: performed experiments. ES, HN, MT, KK, YH, TF: coordinated and oversaw the project. All authors read and approved the final manuscript.
